# P-79. Experience of Bartonella henselae Osteomyelitis in the Pediatric Population at Texas Children’s Hospital: A Retrospective Review from 2004-2025

**DOI:** 10.1093/ofid/ofaf695.308

**Published:** 2026-01-11

**Authors:** Andrea Mendez, Juliana Olson, Claire Bocchini

**Affiliations:** Baylor College of Medicine, Texas Children's Hospital, Houston, TX; Baylor College of Medicine, Houston, Texas; Baylor College of Medicine, Houston, Texas

## Abstract

**Background:**

*Bartonella henselae* is a fastidious, aerobic, intracellular, gram-negative bacillus that causes cat scratch disease (CSD). There is a wide spectrum of presentations of CSD, the most common presentation is localized lymphadenopathy, but disseminated forms can occur reflecting likely hematogenous spread. Osteomyelitis is an unusual presentation of CSD.

**Figure 1:**
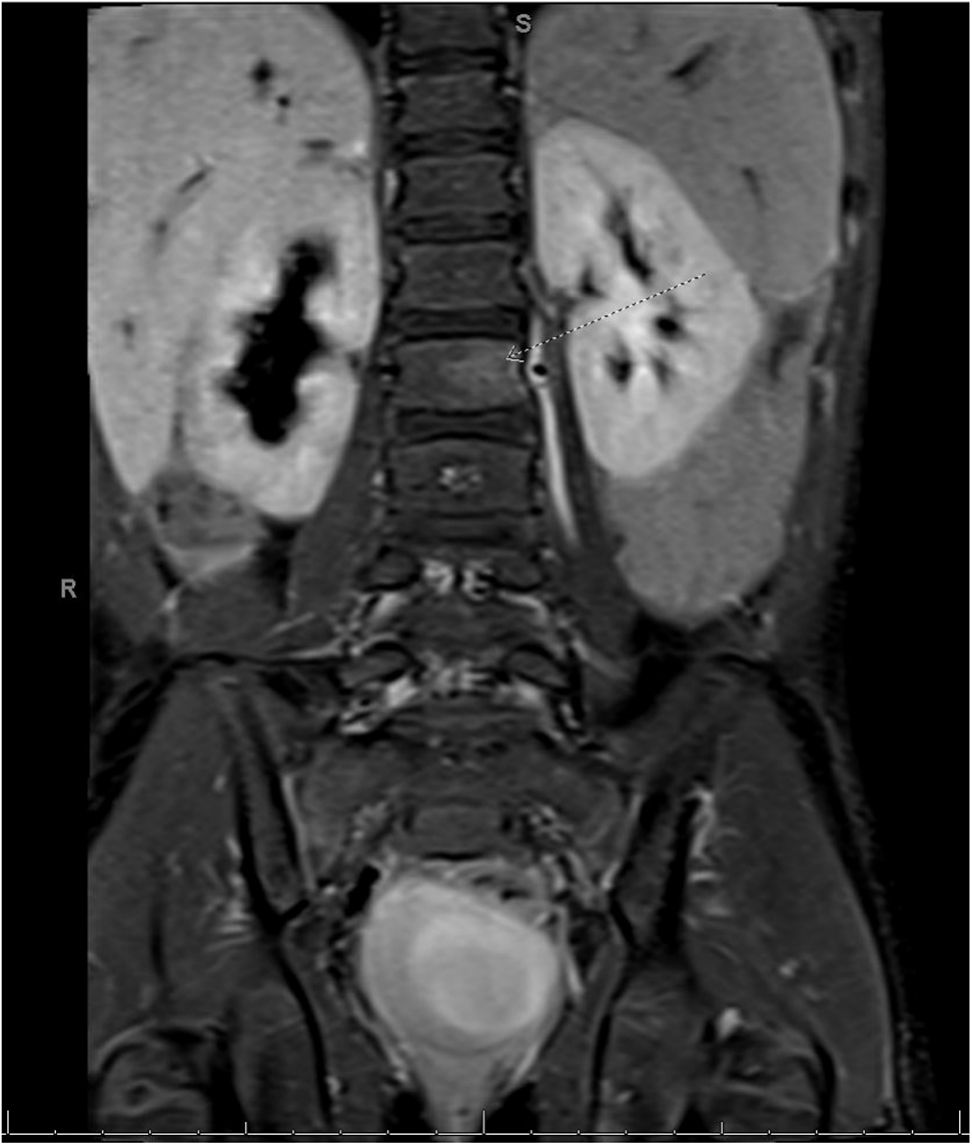
Focal hyperintense signal with abnormal enhancement in the body and pedicle of L2 on the left associated with a small left paraspinal phlegmon centered at L2.

**Figure 2:**
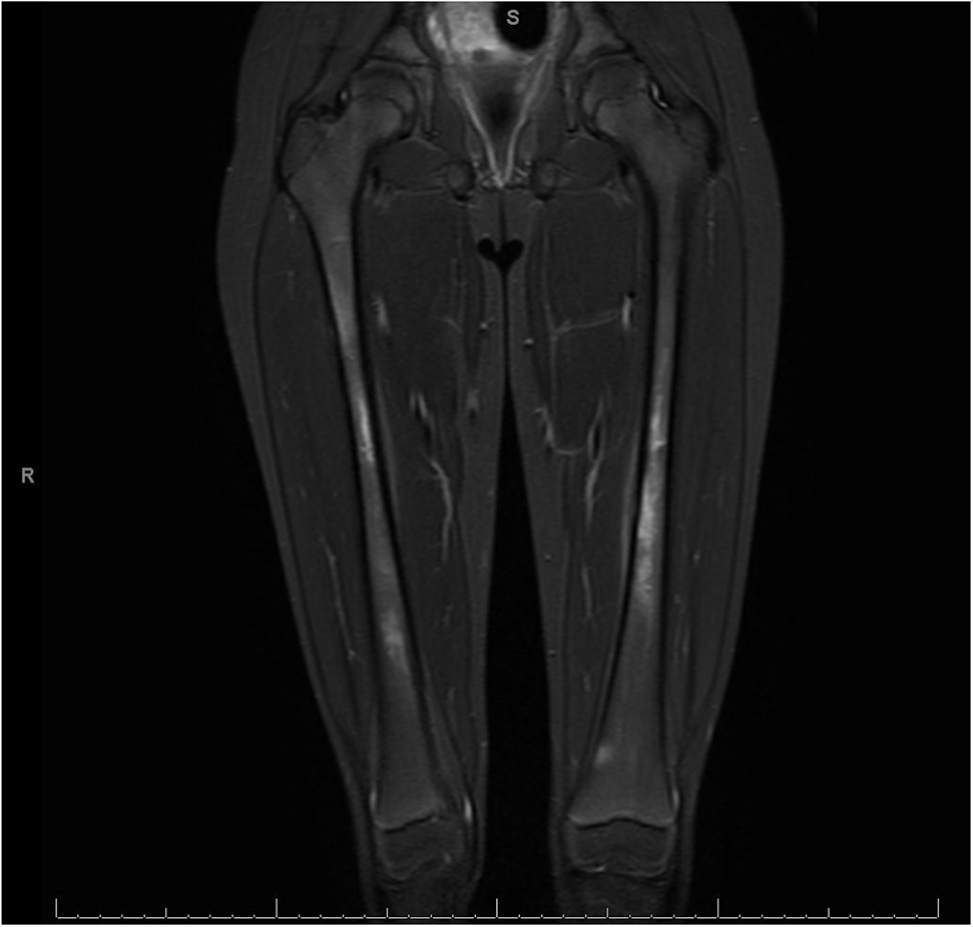
Multiple small foci of eccentric marrow signal abnormality and enhancement with adjacent periostitis in the bilateral femurs.

**Methods:**

This is a retrospective case series from patients from 2004 to 2025 across the Texas Children’s Hospital system. The cohort was identified using ICD9/10 codes for “osteomyelitis” and “bartonellosis” or “cat scratch disease” in both the inpatient and outpatient setting. Diagnosis is established with positive serology, a positive *Bartonella* spp PCR result from tissue, or positive microbial cell-free DNA sequencing (Karius) test for *Bartonella henselae*. Positive serology was defined as probable CSD with patients having IgM >1:16 or IgG 1:64 – 1:256, or definitive CSD defined as IgG >1:256. Osteomyelitis diagnosis was based on magnetic resonance imaging (MRI), clinical presentation, and elevated inflammatory markers.

Table 1. Demographics, exposure, presenting symptoms, diagnosis, and treatment as documented in the medical record

**Figure:**
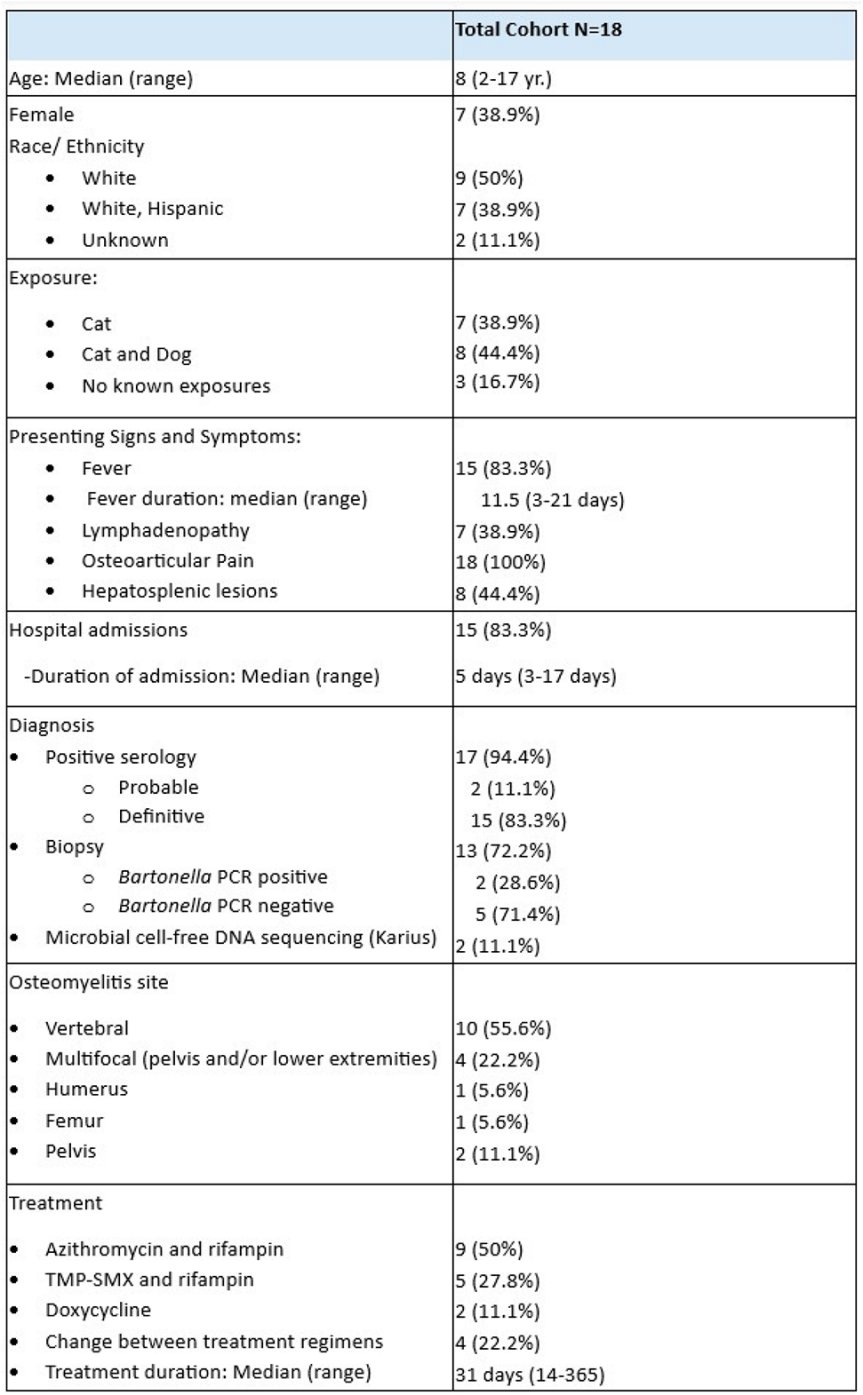

**Results:**

The ICD 9/10 codes identified 26 patients, of which 18 met criteria for *Bartonella henselae* osteomyelitis (Table 1). The median age of presentation was 8 years (n=18, range from 2 to 17 years), with 50% identified as white. All patients presented osteoarticular pain and all underwent MRI (Figures 1, 2). The most common sites for osteomyelitis were vertebral (55.6%) and pelvis (22.2%); 22.2% was multifocal. One patient was diagnosed based solely on Karius while all other patients had positive serology. Of the biopsies undergoing PCR (7), 5 (71.4%) were negative. The most commonly used antibiotic regimen was azithromycin and rifampin (50%), or trimethoprim-sulfamethoxazole and rifampin (27.8%) with a median duration of 31 days. One patient did not receive treatment with subsequent resolution of radiological findings.

**Conclusion:**

Osteomyelitis in cat scratch disease should be suspected in patients with osteoarticular pain and positive serology. Bartonella PCR from tissue biopsy has a low diagnostic yield. More studies are needed to determine preferred treatment and duration.

**Disclosures:**

All Authors: No reported disclosures

